# Polyacrylic Acid-Driven Design of Nd_2_O_3_ Nanostructures for Enhanced Supercapacitor Performance

**DOI:** 10.3390/polym18101194

**Published:** 2026-05-13

**Authors:** Rutuja U. Amate, Aviraj M. Teli, Sonali A. Beknalkar, Chan-Wook Jeon

**Affiliations:** 1 School of Chemical Engineering, Yeungnam University, 280 Daehak-ro, Gyeongsan 38541, Republic of Korea; 2 Division of Electronics and Electrical Engineering, Dongguk University-Seoul, Seoul 04620, Republic of Korea

**Keywords:** rare earth oxide, polyacrylic acid, hydrothermal synthesis, asymmetric supercapacitor, faradaic charge storage, energy storage devices

## Abstract

The rational design of electrode architectures is essential for advancing high-performance supercapacitors. In this study, Nd_2_O_3_ electrodes with controlled structural features were developed via a polyacrylic acid (PAA)-assisted hydrothermal approach. By systematically tuning PAA concentration, the growth mechanism of Nd_2_O_3_ was effectively regulated, leading to a distinct morphological transition from compact agglomerates to well-defined hierarchical structures. The optimized Nd_2_O_3_-P2 electrode exhibits a porous and interconnected architecture, providing enhanced electrolyte accessibility and shortened ion diffusion pathways. This structural optimization significantly improves electrochemical performance, delivering a high areal capacitance of 26.889 F/cm^2^ at 10 mA/cm^2^, along with excellent rate capability and reduced internal resistance. Kinetic analysis reveals that charge storage is predominantly governed by diffusion-controlled Faradaic processes, with the optimized structure facilitating rapid ion transport and efficient redox activity. Additionally, the electrode demonstrates excellent cycling durability, retaining 87.08% capacitance over 12,000 cycles. An asymmetric supercapacitor assembled using Nd_2_O_3_-P2 and activated carbon achieves stable operation up to 1.5 V, delivering good capacitance retention (81.2%) after 7000 cycles. This work highlights the effectiveness of PAA-induced structural tuning and provides a practical strategy for developing advanced rare earth oxide-based electrodes for energy storage applications.

## 1. Introduction

The continuous advancement of portable electronics, wearable devices, and hybrid energy systems has significantly increased the demand for efficient and reliable energy storage technologies [1]. At the same time, the growing use of renewable energy sources has made energy storage even more important, since power generation from these sources is often irregular [2]. This creates a need for systems that can store energy quickly and deliver it when required. In addition, the demand for compact, lightweight, and long-lasting devices has further pushed the development of advanced energy storage solutions [3]. Supercapacitors have emerged as promising candidates due to their rapid charge–discharge capability, high power density, and excellent cycling stability [4]. However, their relatively low energy density compared to battery systems remains a key limitation, largely governed by the intrinsic properties of electrode materials and their ability to store charge efficiently [5]. Therefore, the development of advanced electrode materials with improved electrochemical activity, enhanced conductivity, and efficient ion transport pathways is essential for achieving high-performance energy storage. In recent years, research efforts have expanded beyond conventional transition metal oxides toward alternative material systems [6]. Among these, rare earth oxides have attracted considerable attention owing to their stable chemical nature, unique electronic configurations, and reversible redox behavior [7,8]. Materials such as cerium oxide (CeO_2_), lanthanum oxide (La_2_O_3_), and praseodymium-based oxides have demonstrated promising electrochemical properties; however, their performance is often limited by low electrical conductivity and sluggish ion diffusion, which restrict efficient charge storage [9,10]. Among these, Nd_2_O_3_ represents an interesting yet comparatively less explored rare-earth oxide for electrochemical energy storage. Unlike CeO_2_, which primarily relies on oxygen vacancy-mediated charge storage, or La_2_O_3_, which often exhibits limited redox activity, Nd_2_O_3_ can undergo reversible oxide–hydroxide transformations in alkaline media, enabling Faradaic charge storage. Additionally, Nd-based systems offer good structural stability during repeated redox cycling, which is essential for long-term electrochemical performance. Despite these advantages, the electrochemical behavior of Nd_2_O_3_, particularly in non-composite and polymer-assisted configurations, remains insufficiently investigated. This provides a strong motivation to explore its potential as an active electrode material [7].

In this context, neodymium oxide (Nd_2_O_3_) has emerged as a potential candidate for supercapacitor applications due to its structural stability and redox-active characteristics [11]. Previous studies have mainly focused on combining Nd_2_O_3_with conducting polymers such as polyaniline and polypyrrole to enhance conductivity and electrochemical performance. For instance, Li et al. reported that polyaniline (PANI)/Nd_2_O_3_ composites exhibit higher capacitance than pure PANI due to the increased number of electrochemically active sites, although excessive Nd_2_O_3_ content led to reduced conductivity [12]. Similarly, Abdollahi et al. demonstrated that polypyrrole (PPy)/Nd_2_O_3_ electrodes deliver significantly improved capacitance and good cycling stability compared to pure PPy, confirming the positive role of Nd_2_O_3_ in enhancing faradaic charge storage [13]. Hameed et al. further showed that Nd_2_O_3_ incorporation into PPy nanostructures enhances capacitance, indicating its contribution to improved electrochemical performance [14]. These studies highlight that Nd_2_O_3_ can effectively enhance charge storage; however, the improvements are largely dependent on conductive polymer matrices. In contrast, the use of non-conducting functional polymers remains relatively unexplored. Polyacrylic acid (PAA), although electrically insulating, offers significant advantages due to its abundant carboxyl functional groups, which can interact strongly with metal ions during synthesis [15]. Unlike conducting polymers, PAA does not directly contribute to electrical conductivity but plays a crucial role in regulating nucleation and growth, controlling particle dispersion, and preventing agglomeration. This results in more uniform and porous structures, enhancing electrolyte accessibility and facilitating ion transport. Additionally, PAA improves the adhesion between active material and substrate, contributing to better mechanical stability during cycling [16]. Importantly, the influence of PAA is highly dependent on its concentration, which governs the balance between nucleation control and structural accessibility. At lower concentrations, the limited availability of functional groups results in incomplete surface coordination with Nd^3+^ ions, leading to partial suppression of particle aggregation and non-uniform growth. As the PAA concentration increases, stronger coordination interactions between the carboxyl groups and metal ions effectively regulate nucleation kinetics. This promotes the formation of uniformly distributed and porous nanostructures. This optimized structural arrangement enhances electrolyte penetration and facilitates rapid ion diffusion. However, higher PAA concentration can increase solution viscosity and induce over-coating of the active material surface, which may hinder electron transport and restrict access to electroactive sites. Therefore, a critical balance in polymer concentration is essential to achieve optimal structural and electrochemical performance [17,18]. To further enhance performance, controlling the morphology and surface characteristics of Nd_2_O_3_ through polymer-assisted strategies is essential. Such modifications improve dispersion, increase active sites, and facilitate ion transport, leading to better electrochemical performance. When applied in asymmetric supercapacitors, these optimized materials enable a wider voltage window, resulting in enhanced energy density without sacrificing power capability.

Despite these advantages, systematic investigations on PAA-modified Nd_2_O_3_ remain limited, particularly in understanding the influence of polymer concentration on structural evolution and electrochemical behavior. In this work, Nd_2_O_3_ electrodes modified with controlled concentrations of PAA are synthesized and systematically investigated to clarify the relationship between polymer content and electrochemical performance. Furthermore, the optimized electrode is employed in an asymmetric supercapacitor configuration to demonstrate its practical applicability, providing a rational strategy for the design of non-conducting polymer-assisted rare earth oxide materials. The study also establishes a direct correlation between polymer-assisted structural tuning and device-level performance. These findings offer useful insights for extending similar strategies to other rare earth oxide-based systems.

## 2. Experimental Section

### 2.1. Materials

All chemicals used in this work were of analytical grade and used as received without any further purification. Neodymium nitrate hexahydrate (Nd(NO_3_)_3_·6H_2_O; 99.9% Sigma-Aldrich, St. Louis, MO, USA) was used as the neodymium precursor, while urea (CO(NH_2_)_2_; 99% Sigma-Aldrich, St. Louis, MO, USA) served as a homogeneous precipitating agent. Polyacrylic Acid (PAA (C_3_H_4_O_2_)_n_; 99.9% Sigma-Aldrich, St. Louis, MO, USA; Mol wt. ~450,000) was employed to regulate the growth behavior of the material. Nickel foam was used as the conductive substrate for the direct growth of active material. Deionized (DI) water was used as the solvent throughout the synthesis.

### 2.2. Hydrothermal Synthesis of PAA-Modified Nd_2_O_3_ Electrodes

**Figure 1:**
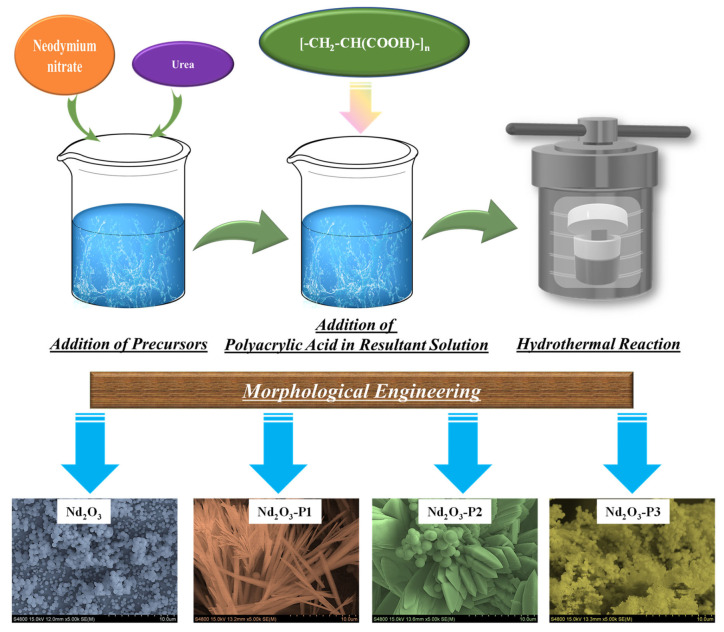
Schematic illustration of the PAA-assisted synthesis pathway for Nd_2_O_3_ electrodes.

PAA-modified Nd_2_O_3_ electrodes were synthesized via a hydrothermal method directly on nickel foam substrates. Prior to synthesis, the nickel foam (1 × 1 cm^2^) was cleaned by ultrasonication in 1 M HCl, ethanol, and DI water successively to remove surface impurities and oxide layers, followed by drying at room temperature. In a typical synthesis, 1.80 g of Nd (NO_3_)_3_·6H_2_O was dissolved in 200 mL of DI water under continuous stirring. Subsequently, 1.50 g of CO(NH_2_)_2_ was added, and the solution was stirred for 30 min. For the modified samples, appropriate amounts of PAA were introduced to obtain concentrations of 0.1%, 0.3%, and 0.5%, followed by further stirring to ensure proper dispersion. The relatively high molecular weight (≈450,000) of PAA plays a significant role in governing the synthesis process. The long polymer chains provide strong steric hindrance and increased solution viscosity, which effectively regulate nucleation and suppress uncontrolled particle growth. This facilitates the formation of more uniform and interconnected nanostructures. Additionally, the abundant carboxyl functional groups along the extended polymer backbone enhance coordination interactions with Nd^3+^ ions, promoting controlled structural evolution. In this study, the molecular weight was kept constant, and the electrochemical behavior was systematically tuned by varying the PAA concentration. The prepared solution was transferred into a Teflon-lined stainless-steel autoclave, and the pre-cleaned nickel foam was immersed vertically in the solution. The autoclave was sealed and maintained at 180 °C for 12 h, and then allowed to cool naturally to room temperature. After completion of the reaction, the coated nickel foam was removed, thoroughly washed with DI water and ethanol to eliminate loosely attached particles, and dried at 80 °C for 12 h. The samples were then annealed at 550 °C for 3 h in air to improve crystallinity and adhesion of the active material. The prepared electrodes were labeled as Nd_2_O_3_ (without PAA), Nd_2_O_3_-P1 (0.1% PAA), Nd_2_O_3_-P2 (0.3% PAA), and Nd_2_O_3_-P3 (0.5% PAA). The schematic illustration of the synthesis of PAA-modified Nd_2_O_3_ electrodes was presented in Figure 1.

### 2.3. Reaction Mechanism of Nd_2_O_3_ Formation

The formation of Nd_2_O_3_ during the hydrothermal and subsequent annealing process can be described through the following reactions. Initially, urea undergoes thermal decomposition, generating hydroxide ions in the solution (1):

(1) $$CO{\left({NH}_{2}\right)}_{2}+{3H}_{2}O\to {2NH}_{4}^{+}+{CO}_{3}^{2-}+{2OH}^{-}$$

The generated hydroxide ions react with Nd^3+^ ions to form neodymium hydroxide (2):

(2) $${Nd}^{3+}+{3OH}^{-}\to {Nd\left(OH\right)}_{3}$$

Upon annealing at elevated temperature, the hydroxide precursor is converted into neodymium oxide (3):

(3) $${2Nd\left(OH\right)}_{3}\to {Nd}_{2}O_{3}+{3H}_{2}O$$

This sequential process enables controlled nucleation and growth of Nd_2_O_3_ on the nickel foam substrate [19,20]. In the presence of optimum PAA concentration, the growth mechanism is further modified through coordination interactions between the carboxyl (–COOH) groups of the polymer and Nd^3+^ ions. These interactions regulate the local supersaturation and act as a soft template, influencing nucleation density and particle growth direction. As a result, PAA-assisted systems exhibit improved structural uniformity and reduced aggregation compared to the pristine sample. Furthermore, the slow conversion of Nd(OH)_3_ to Nd_2_O_3_ during annealing preserves the underlying morphology while enhancing crystallinity. This combination of controlled nucleation, polymer-assisted growth, and thermal transformation plays a critical role in determining the final architecture and electrochemical properties of the electrode material [18].

### 2.4. Sample Characterization and Electrochemical Measurements

The phase composition and crystallinity of Nd_2_O_3_, Nd_2_O_3_-P1, Nd_2_O_3_-P2, and Nd_2_O_3_-P3 electrodes were analyzed using X-ray diffraction (XRD, PANalytical, Almelo, The Netherlands) with Cu Kα radiation to confirm the formation of crystalline Nd_2_O_3_ and examine the influence of PAA concentration. The diffraction patterns were further used to assess phase purity and any structural variation induced by PAA incorporation. The surface morphology of the samples grown on nickel foam was studied using FE-SEM (S-4800, Hitachi, Tokyo, Japan). A thin platinum coating was applied prior to imaging to reduce charging effects. The observations reveal clear morphological evolution with increasing PAA content, indicating its role in tuning surface architecture. Energy-dispersive X-ray spectroscopy (EDS) was used to confirm the presence and uniform distribution of Nd and O elements across the electrodes. X-ray photoelectron spectroscopy (XPS, K-Alpha, Thermo Scientific, Cheshire, UK) was employed to analyze the surface chemical states of Nd and O, providing insight into their oxidation states and surface composition. Electrochemical measurements were carried out using a Biologic WBCS3000 workstation in a three-electrode configuration (BioLogic Science Instruments, Seyssinet-Pariset, France), where the prepared electrodes on nickel foam served as the working electrode, platinum as the counter electrode, and Ag/AgCl as the reference electrode in 2 M KOH electrolyte. Cyclic voltammetry (CV) and galvanostatic charge–discharge (GCD) tests were performed at different scan rates and current densities to evaluate capacitive behavior and rate capability. Electrochemical impedance spectroscopy (EIS) was conducted to study charge transfer resistance and ion diffusion, while cycling tests were used to assess long-term stability. The areal capacitance values were calculated from GCD curves to ensure accurate evaluation of electrochemical performance.

## 3. Results and Discussions

### 3.1. XRD Elucidation

**Figure 2:**
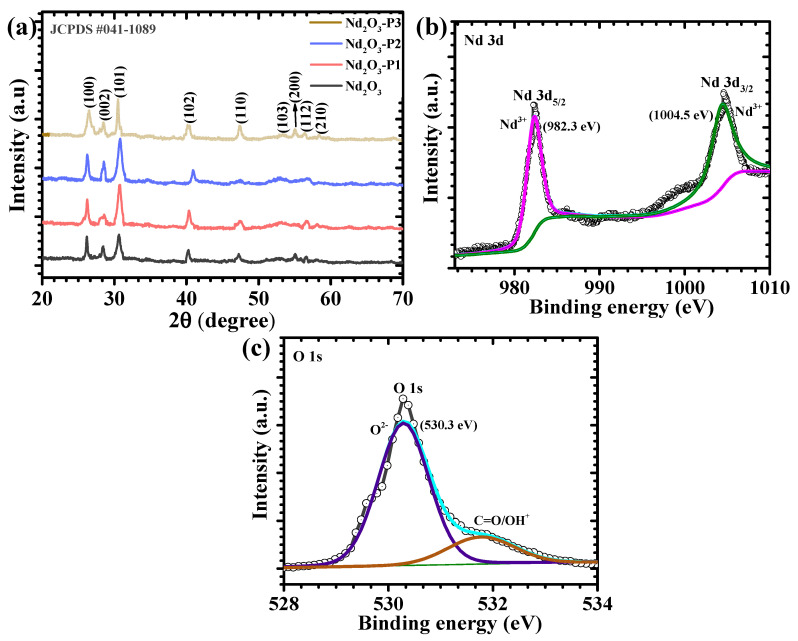
(**a**) XRD patterns of pristine Nd_2_O_3_ and PAA-modified samples (Nd_2_O_3_-P1, Nd_2_O_3_-P2, Nd_2_O_3_-P3), High-resolution XPS spectra of the optimized Nd_2_O_3_-P2 electrode (**b**) Nd 3d, and (**c**) O 1s.

The crystal structure of Nd_2_O_3_, Nd_2_O_3_-P1, Nd_2_O_3_-P2, and Nd_2_O_3_-P3 was investigated using X-ray diffraction, as shown in Figure 2a. All samples display distinct diffraction peaks at 2θ values of 26.51°, 28.48°, 30.57°, 40.36°, 47.48°, 53.36°, 55.66°, 56.53°, and 58.45°, which correspond to the (100), (002), (101), (102), (110), (103), (200), (112), and (210) planes of hexagonal Nd_2_O_3_ (JCPDS #041-1089). These reflections clearly confirm the formation of crystalline Nd_2_O_3_ across all samples. No extra diffraction peaks are observed, indicating that the samples are free from secondary phases and that the addition of PAA does not alter the crystal structure of Nd_2_O_3_. However, variations in peak intensity and width can be observed among the samples. The Nd_2_O_3_ sample exhibits relatively broad and less intense peaks, suggesting lower crystallinity. With the introduction of PAA, a noticeable increase in peak intensity and sharpness is observed. This effect is most prominent in Nd_2_O_3_-P2, where the diffraction peaks become sharper and more intense, indicating improved crystallinity and better structural ordering. This suggests that an appropriate amount of PAA facilitates controlled nucleation and growth during synthesis. In contrast, the Nd_2_O_3_-P3 sample shows a slight reduction in peak intensity along with some peak broadening, indicating that higher PAA may hinder proper crystal growth and introduce structural disorder. Despite these differences, the peak positions remain unchanged for all samples, confirming that PAA mainly influences crystallinity and microstructural features without affecting the phase composition [21].

### 3.2. XPS Analysis

XPS was performed on the optimized Nd_2_O_3_-P2 sample to examine its surface chemical composition and oxidation states. The high-resolution Nd 3d spectrum (Figure 2b) shows two well-defined peaks located at approximately 982.3 eV and 1004.5 eV, corresponding to Nd 3d_5/2_ and Nd 3d_3/2_, respectively. These binding energies are characteristic of Nd^3+^, confirming the presence of neodymium in its trivalent state within Nd_2_O_3_. The absence of additional peaks related to other oxidation states indicates the formation of a chemically stable and pure oxide phase [22]. The O 1s spectrum (Figure 2c) exhibits a dominant peak centered around 530.3 eV, which is attributed to lattice oxygen in Nd_2_O_3_. A secondary component observed at 531.9 eV is associated with surface hydroxyl groups or adsorbed oxygen species. These surface species are commonly present in oxide materials and can improve surface wettability, thereby facilitating better interaction with the electrolyte. The well-defined peak positions and their clear separation indicate a stable chemical environment and good surface uniformity of the Nd_2_O_3_-P2 sample [23]. The coexistence of lattice oxygen and a small amount of surface hydroxyl groups may provide active sites for electrochemical reactions and support efficient ion diffusion. In addition, the absence of noticeable peak shifts suggests that there is no significant lattice distortion or change in the electronic structure of Nd_2_O_3_ after PAA incorporation. The relatively narrow peak profiles further indicate good crystallinity at the surface level, which is consistent with the XRD observations.

### 3.3. Morphological and Elemental Composition

**Figure 3:**
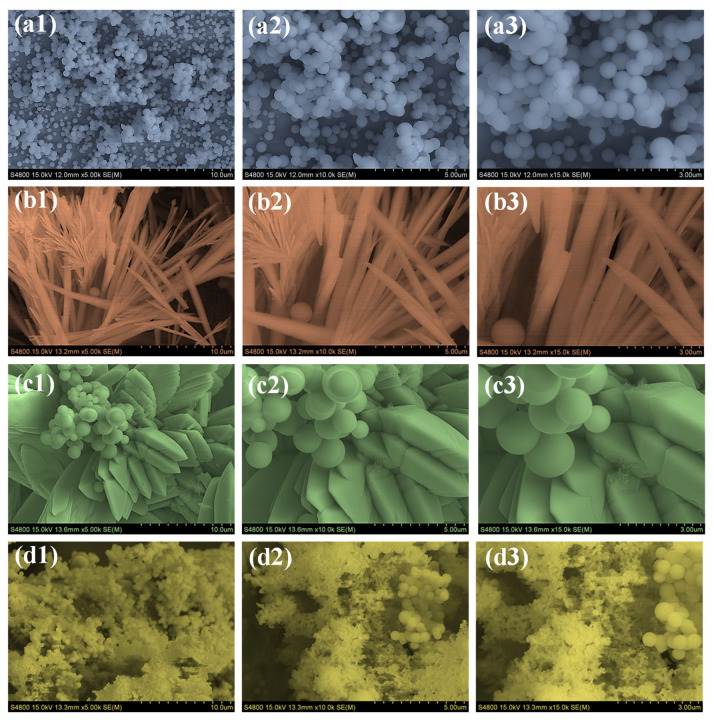
FESEM images showing morphological evolution of (**a1**–**a3**) Nd_2_O_3_, (**b1**–**b3**) Nd_2_O_3_-P1, (**c1**–**c3**) Nd_2_O_3_-P2, and (**d1**–**d3**) Nd_2_O_3_-P3 at different magnifications.

The surface morphology of the synthesized Nd_2_O_3_ samples with varying PAA concentrations was examined using FESEM. The bare Nd_2_O_3_ sample consists of densely packed, highly agglomerated spherical nanoparticles, as shown in Figure 3(a1–a3). The particles are closely clustered with no clear structural organization, indicating uncontrolled nucleation and growth in the absence of a structure-directing agent. With the addition of PAA, distinct morphological changes are observed. Nd_2_O_3_-P1 exhibits the formation of elongated rod-like structures alongside residual spherical particles, as seen in Figure 3(b1–b3). These rods are randomly oriented and densely distributed, suggesting that a low PAA concentration promotes anisotropic growth while some agglomeration remains. This indicates that PAA begins to influence particle formation by altering the nucleation and growth kinetics. The more pronounced transformation occurs in Nd_2_O_3_-P2, where well-defined hierarchical architectures are clearly visible in Figure 3(c1–c3). The images show a combination of plate-like structures and spherical particles arranged in organized, flower-like assemblies. This interconnected framework provides a more open structure with higher surface accessibility. The reduced agglomeration and uniform particle distribution demonstrate effective control over growth at this optimized PAA concentration, creating a morphology favorable for electrochemical applications due to enhanced ion transport and electrolyte penetration. In contrast, further increasing the PAA concentration in Nd_2_O_3_-P3 leads to a decline in structural uniformity, as seen in Figure 3(d1–d3). The morphology becomes irregular and highly agglomerated, forming dense clusters with a loss of well-defined architecture. Higher PAA likely over-coordinates with Nd ions, hindering controlled crystal growth and causing particle accumulation. These observations show a clear progression in morphology with increasing PAA content: the nanoparticles in Nd_2_O_3_ gradually evolve into rod-like structures in Nd_2_O_3_-P1, develop into well-organized, flower-like assemblies in Nd_2_O_3_-P2, and become irregular and densely clustered in Nd_2_O_3_-P3. The observed morphological evolution with increasing PAA concentration indicates that the polymer plays a critical role in regulating particle growth and dispersion, leading to a more open and interconnected architecture in the optimized sample. Such structural features are expected to enhance electrolyte penetration and increase the effective electroactive surface area.

**Figure 4:**
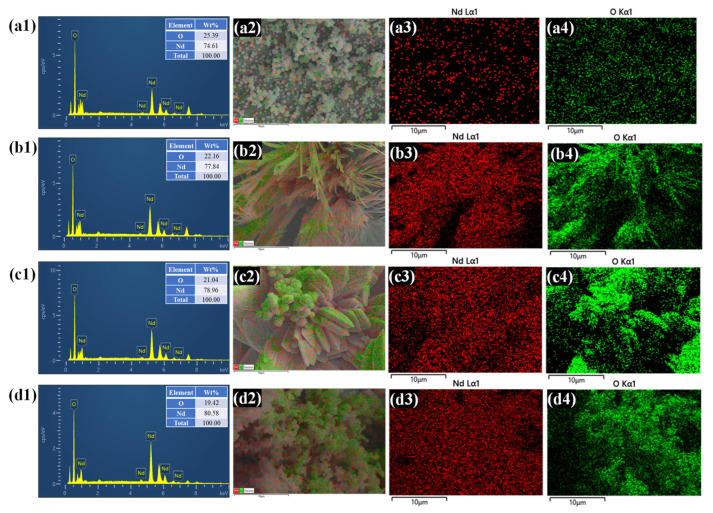
EDS analysis and elemental mapping of (**a1**–**a4**) Nd_2_O_3_, (**b1**–**b4**) Nd_2_O_3_-P1, (**c1**–**c4**) Nd_2_O_3_-P2, and (**d1**–**d4**) Nd_2_O_3_-P3.

The elemental composition and spatial distribution of Nd_2_O_3_, Nd_2_O_3_-P1, Nd_2_O_3_-P2, and Nd_2_O_3_-P3 electrodes were examined using EDS and elemental mapping with corresponding FESEM layered image. The EDS spectra (Figure 4(a1–d1)) confirm the presence of Nd and O as the primary constituents in all samples, verifying the successful formation of the oxide phase without detectable impurities. A gradual increase in Nd content, accompanied by a slight decrease in oxygen content, is observed from Nd_2_O_3_ to Nd_2_O_3_-P3, which can be attributed to improved crystallinity and densification with increasing PAA concentration. Elemental mapping further highlights the evolution in distribution characteristics. The pristine Nd_2_O_3_ sample (Figure 4(a2–a4)) exhibits non-uniform elemental dispersion, consistent with its agglomerated morphology. In contrast, Nd_2_O_3_-P1 (Figure 4(b2–b4)) and Nd_2_O_3_-P2 (Figure 4(c2–c4)) display a more homogeneous and continuous distribution, indicating that moderate incorporation of PAA effectively enhances particle dispersion and surface coverage. Notably, Nd_2_O_3_-P2 demonstrates the most uniform elemental distribution, correlating with its optimized structural features. However, a slight degree of aggregation and localized compositional variation is observed in Nd_2_O_3_-P3 (Figure 4(d2–d4)), suggesting that higher PAA begins to compromise structural uniformity.

### 3.4. Electrochemical Analysis

**Figure 5:**
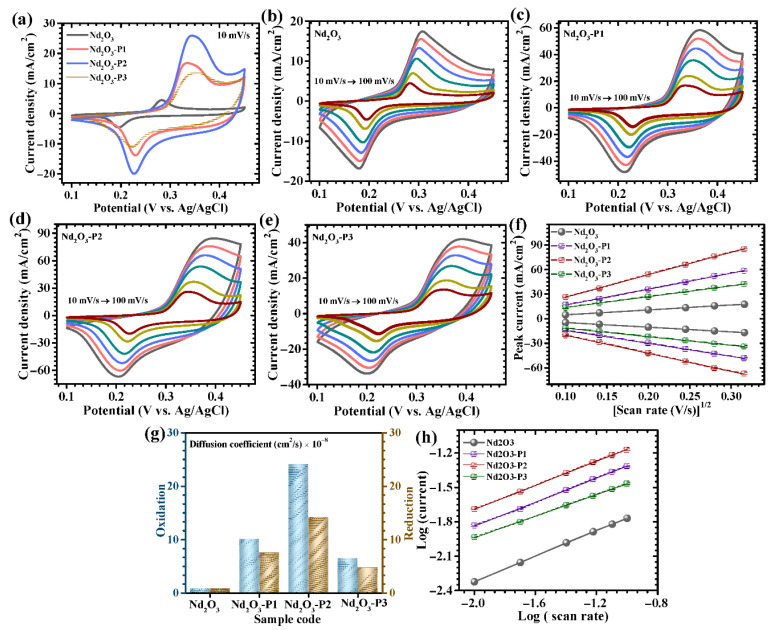
Cyclic voltammetry evaluation of Nd_2_O_3_-based electrodes: (**a**) CV curves at a fixed scan rate (10 mV/s), (**b**–**e**) Scan rate-dependent CV profiles for all electrodes (10–100 mV/s), (**f**) Variation in peak current with square root of scan rate, (**g**) Comparative diffusion coefficient analysis, and (**h**) Determination of b-values from *log*(*i*)–*log*(*v*) plots.

The electrochemical behavior of the as-prepared Nd_2_O_3_ electrodes, synthesized with systematically varied PAA concentrations, was thoroughly examined to establish the influence of polymer-assisted structural regulation on charge storage performance. The incorporation of PAA plays a decisive role in modulating nucleation dynamics, surface functionality, and microstructural organization, which collectively govern the electrochemical response. CV measurements performed in a potential window 0.1 to 0.45 V versus Ag/AgCl at a scan rate of 10 mV/s (Figure 5a) reveal that all electrodes exhibit distinct anodic and cathodic features, indicating that the charge storage mechanism is predominantly governed by surface-controlled Faradaic reactions. These redox signatures are associated with reversible transitions of neodymium species, facilitated through interaction with hydroxide ions in the alkaline electrolyte. A clear dependence of electrochemical response on PAA concentration is observed. The pristine Nd_2_O_3_ electrode displays relatively weak and broadened redox features with limited current response, suggesting sluggish charge transfer kinetics and restricted electrochemically active surface area. This behavior can be attributed to particle agglomeration and dense morphology formed in the absence of a structure-directing agent. Upon introducing a low concentration of PAA (Nd_2_O_3_-P1), a noticeable enhancement in peak definition and current intensity is achieved. This improvement indicates better dispersion of primary particles and partial suppression of aggregation, leading to increased accessibility of active sites [24]. The electrode synthesized with an intermediate PAA concentration (Nd_2_O_3_-P2) demonstrates the most prominent redox peaks along with the largest enclosed CV area, signifying superior electrochemical activity. This optimized performance arises from the multifunctional role of PAA, where its carboxyl functional groups coordinate with Nd^3+^ ions. This regulating crystal growth and promoting the formation of a loosely packed, porous architecture [25]. Such a structure provides shortened ion diffusion pathways, enhanced electrode–electrolyte interaction, and improved charge transfer kinetics. In contrast, further increasing the PAA concentration (Nd_2_O_3_-P3) results in a decline in electrochemical response. Higher polymer content can lead to increased solution viscosity and excessive surface coverage, which may partially block electroactive sites and impede electron transport pathways. Consequently, the balance between structural porosity and electrical conductivity is disrupted, leading to reduced performance [26]. The redox profiles of all electrodes exhibit quasi-symmetric characteristics, indicative of reversible electrochemical processes. Notably, the Nd_2_O_3_-P2 electrode shows the highest degree of symmetry with minimal peak separation, reflecting reduced polarization and enhanced reaction reversibility. The charge storage behavior of the Nd_2_O_3_ electrode is predominantly governed by Faradaic processes involving hydroxide ion interaction in alkaline electrolyte. Based on established literature reports on rare-earth oxide systems, this behavior can be described by the following reversible reactions (4) and (5) [27,28]:

(4) $${Nd}_{2}O_{3}+H_{2}O⇌\;2NdOOH$$

(5) $$2NdOOH+{OH}^{-}⇌\;Nd{\left(OH\right)}_{2}+e^{-}$$

These reactions represent a possible redox pathway involving reversible hydroxide-ion-assisted transformation of Nd species. The presence of distinct redox features in CV profiles collectively supports a Faradaic charge storage mechanism in the present system. The incorporation of PAA significantly enhances this redox activity by tailoring the local coordination environment and inducing defect-rich surfaces, which serve as additional electroactive centers. The effect of scan rate on electrochemical behavior was further investigated over a range of 10–100 mV/s (Figure 5b–e). With increasing scan rate, all electrodes show a proportional increase in current response while largely preserving the shape of the CV curves, confirming robust electrochemical stability and fast redox kinetics. A slight shift in peak positions at higher scan rates is observed due to internal resistance and polarization effects [29]; however, this shift is significantly less pronounced for the Nd_2_O_3_-P2 electrode, indicating faster charge transfer and superior rate capability. The ability to retain well-defined redox characteristics at elevated scan rates underscores the efficient ion transport and structural integrity of the optimized electrode.

A detailed kinetic investigation of the Nd_2_O_3_-based electrodes was carried out to elucidate the interplay between redox dynamics and ion transport as a function of PAA concentration. CV performed over an extended range of scan rates reveals that both anodic and cathodic peak currents (*i_p_*) exhibit a well-defined linear relationship with the square root of the scan rate (*v*^1/2^) for all samples (Figure 5f), indicating that the charge storage process is predominantly governed by diffusion-controlled Faradaic reactions. This behavior reflects the involvement of reversible redox transformations facilitated by efficient hydroxide ion transport within the electrode matrix. To quantitatively assess ion diffusion characteristics, the apparent diffusion coefficients (D) were determined using the Randles–Sevcik relationship, expressed as (6) [30]:

(6) $$D=\frac{i_{p}}{2.69\;\times \;10^{5}\;\times \;n^{3/2}\;\times \;A\;\times \;C\;\times \;v^{1/2}}$$

where *n* denotes the number of electrons transferred, *A* represents the effective electroactive area, *C* is the concentration of redox-active species, and *v* is the scan rate. The extracted diffusion coefficients at 10 mV/s are summarized in Table 1 and comparatively illustrated in Figure 5g. Among the investigated electrodes, the Nd_2_O_3_-P2 sample consistently delivers the highest diffusion coefficient of 24.047 × 10^−8^ (Oxidation) and 14.184 × 10^−8^ (reduction), signifying markedly enhanced ionic mobility and accelerated charge propagation kinetics. This superior performance is intrinsically associated with the optimized role of PAA, which governs nucleation and growth through metal–polymer coordination, ultimately yielding a porous, defect-rich, and well-interconnected framework. Such an architecture facilitates unobstructed electrolyte infiltration, maximizes the exposure of electroactive sites, and shortens ion diffusion pathways, thereby promoting rapid Faradaic reactions. In contrast, the Nd_2_O_3_ electrode synthesized without PAA exhibits comparatively lower diffusion coefficients due to its compact and agglomerated structure, which limits ion accessibility and slows charge transfer. The Nd_2_O_3_-P1 electrode shows moderate improvement, reflecting partial structural refinement and improved dispersion of active material. However, higher incorporation of PAA in Nd_2_O_3_-P3 leads to a decline in diffusion behavior, which can be attributed to partially obstructed active sites and impeded ion transport channels. Consequently, the optimized Nd_2_O_3_-P2 electrode achieves an ideal balance between structural openness and electronic connectivity, enabling efficient ion diffusion and enhanced electrochemical kinetics.

To further elucidate the underlying charge storage mechanism, a quantitative analysis of the relationship between peak current (*i*) and scan rate (*v*) was performed using the power-law expression (7) [31]:

(7) $$i={av}^{b}$$

where the exponent *b* serves as a critical descriptor of the dominant electrochemical process. In general, b values approaching 0.5 are indicative of diffusion-controlled Faradaic reactions, whereas values nearing 1 correspond to surface-confined capacitive behavior. The b-values for all Nd_2_O_3_-based electrodes were determined from the slope of linear fits to the *log*(*i*) *versus log*(*v*) plots (Figure 5h), and the extracted values are compiled in Table 1. Notably, the resultant b-values for the Nd_2_O_3_, Nd_2_O_3_-P1, Nd_2_O_3_-P2, and Nd_2_O_3_-P3 electrodes lie within the range of approximately 0.47–0.56, confirming that the charge storage process is predominantly governed by diffusion-controlled redox reactions involving hydroxide ion intercalation and bulk Faradaic transformations. Importantly, the Nd_2_O_3_-P2 electrode exhibits a *b*-value closer to 0.5 compared to its counterparts, suggesting an enhanced contribution from diffusion-controlled processes.

A more resolved interpretation of the charge storage dynamics was achieved by deconstructing the current response into its intrinsic kinetic components using the relation (8) [32,33], which enables clear differentiation between surface-controlled capacitive processes and diffusion-governed Faradaic contributions.

(8) $$i\left(V\right)=k_{1}v{+k_{2}v}^{1/2}$$

**Figure 6:**
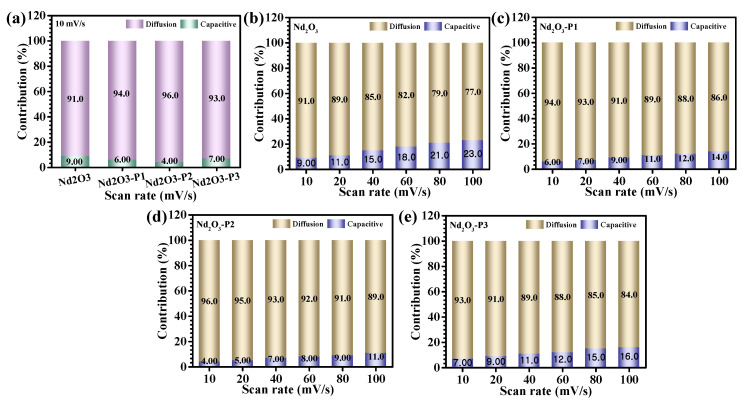
Charge storage kinetics analysis: (**a**) Relative contribution of diffusion-controlled and capacitive processes at low scan rate, (**b**–**e**) Evolution of capacitive and diffusion contributions with increasing scan rate for Nd_2_O_3_ and PAA-modified electrodes.

In this framework, the *k_1_v* term represents the capacitive component associated with rapid charge accumulation at the electrode–electrolyte interface, whereas the *k*_2_*v*^1/2^ term corresponds to diffusion-limited processes involving ion insertion and bulk redox transformations. The proportionality constants were extracted through linear fitting of *i*(*V*)/*v*^1/2^ versus *v*^1/2^, allowing a reliable deconvolution of the two contributions. Accordingly, the total stored charge (*Q_t_*) can be expressed as the sum of surface capacitive (*Q_s_*) and diffusion-controlled (*Q_d_*) components, i.e., *Q_t_* = *Q_s_* + *Q_d_*. Analysis performed at a low scan rate (10 mV/s) reveals that diffusion-controlled processes overwhelmingly dominate the charge storage behavior across all electrodes, confirming that bulk Faradaic activity is the principal mechanism (Figure 6a). Notably, the Nd_2_O_3_-P2 electrode exhibits the highest diffusion contribution (96%) among the series, indicating a more effective utilization of the electroactive bulk and significantly enhanced ion transport kinetics. This behavior can be directly linked to the optimized role of PAA, which induces a structurally open and interconnected framework through controlled metal–polymer coordination, thereby facilitating deeper electrolyte penetration and activating a larger fraction of redox-active sites. Furthermore, the evolution of charge storage characteristics with increasing scan rate reveals a progressive increase in the capacitive contribution for all electrodes (Figure 6b–e), which can be attributed to the reduced time available for ions to diffuse into the interior at higher sweep rates, thereby favoring surface-dominated processes [34]. Despite this inherent kinetic shift, the Nd_2_O_3_-P2 electrode consistently retains a comparatively higher diffusion contribution across the entire scan rate range, highlighting its superior ion transport capability and structural robustness. This ability to sustain bulk redox activity even under dynamic conditions reflects an optimal balance between diffusion pathways and interfacial reactivity, ultimately enabling efficient charge storage under both low- and high-rate operation.

**Figure 7:**
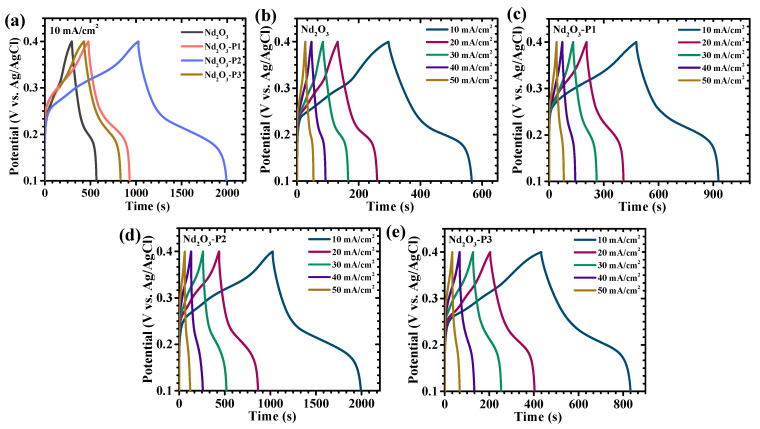
Galvanostatic charge–discharge behavior of Nd_2_O_3_ electrodes: (**a**) GCD profiles at a fixed current density (10 mA/cm^2^), and (**b**–**e**) Charge–discharge curves at different current densities (10–50 mA/cm^2^).

**Figure 8:**
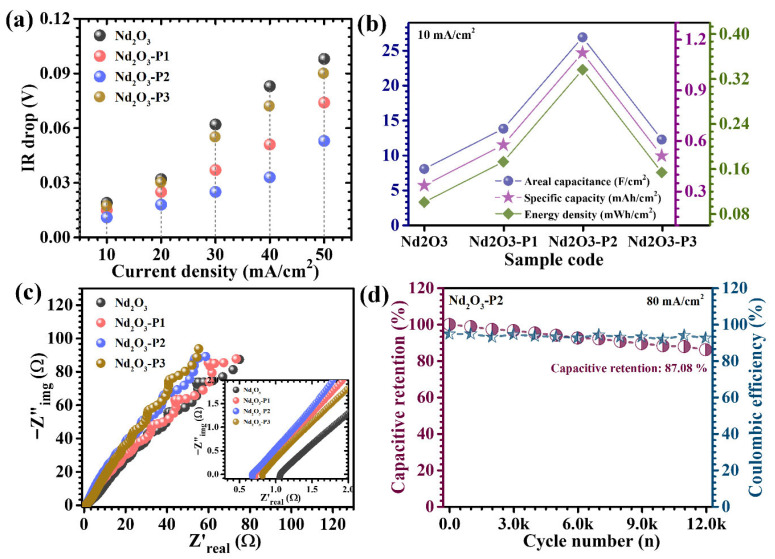
(**a**) Variation in IR drop, (**b**) Energy storage parameters as a function of current density, (**c**) Nyquist plots, and (**d**) Long-term cycling stability of the optimized Nd_2_O_3_-P2 electrode for 12,000 GCD cycles.

The GCD characteristics of the Nd_2_O_3_-based electrodes were systematically evaluated to probe their charge storage behavior and kinetic response as a function of PAA concentration. The GCD profiles recorded at a potential window of 0.1 to 0.4 V versus Ag/AgCl at 10 mA/cm^2^ (Figure 7a) exhibit distinctly non-linear charge–discharge curves with well-defined potential plateaus, confirming that the energy storage mechanism is predominantly governed by Faradaic redox reactions rather than ideal electric double-layer capacitance. These plateaus are associated with reversible neodymium redox transitions mediated through hydroxide ion interaction within the electrolyte. A comprehensive analysis over a wide current density range (10–50 mA/cm^2^) (Figure 7b–e) reveals a clear compositional dependence, where the Nd_2_O_3_-P2 electrode demonstrates a significantly prolonged discharge time compared to Nd_2_O_3_, Nd_2_O_3_-P1, and Nd_2_O_3_-P3, indicating superior charge storage capability and enhanced utilization of electroactive sites. The relatively smooth and gradual voltage decay observed for Nd_2_O_3_-P2 further signifies stabilized redox processes with reduced polarization losses, whereas the pristine Nd_2_O_3_ electrode exhibits shorter discharge durations due to its compact and agglomerated morphology, which limits ion diffusion and active site accessibility. In contrast, the Nd_2_O_3_-P3 electrode shows diminished performance, attributed to higher PAA-induced surface coverage that restricts electron transport and partially blocks electroactive regions. The high degree of symmetry between the charge and discharge curves across all electrodes confirms good coulombic efficiency and reversibility; however, the IR drop (ΔV) at the beginning of discharge provides deeper insight into internal resistance characteristics. The Nd_2_O_3_-P2 electrode exhibits the smallest IR drop (Figure 8a), indicating lower equivalent series resistance (ESR) and improved electronic conductivity, which can be attributed to its optimized interconnected and porous framework [35]. As the current density increases, the IR drop progressively increases for all electrodes due to enhanced polarization effects, yet Nd_2_O_3_-P2 consistently maintains the lowest voltage drop, highlighting its superior rate capability [36,37].

For quantitative evaluation, the areal capacitance (C_A_), energy density (ED), and power density (PD) were calculated using an integrated approach suitable for non-linear Faradaic systems, expressed as (9)–(11) [38,39]:

(9) $$C_{A}=\frac{I\;\times \;2\;\times \;\int V\left(t\right)dt}{A\;\times \;{\left(dV\right)}^{2}}$$

(10) $$ED=\frac{1}{2\times 3600}\;C_{A}\times {dV}^{2}$$

(11) $$PD=\frac{ED\times 3600}{td}$$

where *I* is the discharge current (varied systematically), *∫V*(*t*)*dt* represents the integrated area under the discharge curve (full discharge region to accurately account for the non-linear behavior associated with Faradaic reactions), *A* is the electrode area (1 × 1 cm^2^), *dV* is the operating potential window, and *td* is the discharge time. This methodology ensures accurate determination of capacitance by accounting for the full contribution of Faradaic reactions. It should be noted that the electrochemical performance in the present work is evaluated primarily on an areal basis due to the binder-free in situ growth of active material on nickel foam. In such substrate-integrated systems, accurate determination of the deposited active mass through weight difference may involve considerable uncertainty owing to possible substrate changes during hydrothermal treatment and annealing. Therefore, areal capacitance provides a more reliable representation of electrode performance. The calculated values (Table 2) reveal a strong dependence on PAA concentration, with the Nd_2_O_3_-P2 electrode delivering the highest areal capacitance of 26.889 F/cm^2^ at 10 mA/cm^2^ current density (Figure 8b), significantly outperforming Nd_2_O_3_, Nd_2_O_3_-P1, and Nd_2_O_3_-P3. This superior performance arises from the synergistic combination of enhanced electroactive surface area, improved electrolyte accessibility, and accelerated ion diffusion pathways enabled by optimal polymer incorporation. Furthermore, rate capability analysis demonstrates a gradual decrease in capacitance with increasing current density for all electrodes, primarily due to limited ion diffusion into deeper regions at higher rates. However, Nd_2_O_3_-P2 exhibits markedly improved capacitance retention, maintaining a substantial fraction of its initial capacitance even under high current densities, which underscores its ability to sustain efficient charge storage under rapid cycling conditions. This enhanced rate performance is attributed to reduced diffusion resistance, improved electrical conductivity, and shortened ion transport pathways. Overall, these results confirm that precise tuning of PAA concentration establishes an optimal balance between structural porosity, electronic conductivity, and redox activity, thereby enabling the Nd_2_O_3_-P2 electrode to achieve superior electrochemical performance compared to its counterparts.

Electrochemical impedance spectroscopy (EIS) was employed to unravel the intrinsic charge transfer characteristics and ion transport behavior of the Nd_2_O_3_-based electrodes. The Nyquist plots (Figure 8c) exhibit the characteristic impedance response comprising a high-frequency intercept on the real axis, a semicircle in the intermediate frequency region, and a nearly linear Warburg tail in the low-frequency domain. The high-frequency intercept corresponds to the equivalent series resistance (Rs), which integrates contributions from the intrinsic resistance of the active material, ionic resistance of the electrolyte, and interfacial contact resistance between the electrode and current collector [40]. The semicircular feature reflects the charge transfer resistance, directly associated with the kinetics of Faradaic reactions occurring at the electrode–electrolyte interface, while the inclined line at lower frequencies represents the diffusion-controlled impedance arising from ion transport within the electrode matrix [41]. A pronounced variation in impedance response is observed with PAA incorporation. Among all electrodes, the Nd_2_O_3_-P2 sample exhibits the smallest semicircle diameter, indicating the lowest charge transfer resistance and thus the most favorable electron transfer kinetics. Furthermore, the Rs value extracted for Nd_2_O_3_-P2 is the lowest (0.67 Ω) among the series (Table 1), signifying reduced internal resistance and improved electrical conductivity, further confirming improved interfacial charge transfer kinetics, which can be directly correlated with its structurally interconnected morphology that promotes efficient electron transport and electrolyte interaction.

The long-term electrochemical stability of the optimized Nd_2_O_3_-P2 electrode was further evaluated through extended GCD cycling of 12,000 cycles at a high current density of 80 mA/cm^2^ (Figure 8d). The electrode demonstrates remarkable durability, retaining a substantial percentage of its initial capacitance (87.08%) even after prolonged cycling, along with consistently high coulombic efficiency (92.53%), indicative of highly reversible redox processes and minimal energy dissipation. This excellent stability can be ascribed to the structural resilience imparted by PAA-assisted synthesis, which enables the formation of a mechanically robust and hierarchically organized framework. Such a structure effectively accommodates the repeated volumetric changes associated with hydroxide ion insertion and extraction during cycling, thereby mitigating structural degradation. The interconnected and porous architecture not only preserves electrical continuity but also prevents the collapse or detachment of active material from the substrate [42]. The slight capacitance decay observed over extended cycling may be associated with gradual ion trapping within microstructural domains, minor surface reconstruction induced by continuous redox activity, or partial blockage of electroactive sites [43]. However, the relatively small magnitude of this decline confirms that the electrode retains its structural integrity and electrochemical functionality to a significant extent. Moreover, the sustained high coulombic efficiency throughout the cycling process suggests negligible side reactions and a stable electrode–electrolyte interface. Collectively, these results demonstrate that the optimized PAA incorporation not only enhances charge transfer and ion diffusion but also significantly improves the long-term durability of Nd_2_O_3_-P2 electrodes, making it a promising candidate for reliable high-performance supercapacitor applications.

To further evaluate the significance of the present work, a comparative analysis with previously reported Nd_2_O_3_-based and rare-earth oxide electrode systems is summarized in Table 3 [13,28,33,42,44,45]. Direct comparison should be interpreted cautiously due to differences in electrode configuration and normalization basis (areal vs. gravimetric capacitance). Representative studies, including Nd_2_O_3_-hybrid structures, demonstrate that enhanced electrochemical performance is typically achieved through multi-component compositing or synergistic coupling between different redox-active phases. While these strategies are effective, they often involve increased synthetic complexity and may obscure the intrinsic contribution of the primary active material. In contrast, the present study adopts a fundamentally different approach based on polymer-assisted structural modulation, where PAA governs nucleation and growth behavior to produce a structurally optimized Nd_2_O_3_ electrode. The resulting architecture facilitates improved electrolyte accessibility, enhanced ion diffusion pathways, and better utilization of electroactive sites without introducing additional electrochemically active components. As a result, the optimized Nd_2_O_3_-P2 electrode delivers competitive electrochemical performance compared to previously reported composite systems. These findings highlight that controlled morphological engineering alone can play a decisive role in enhancing charge storage characteristics, offering a simpler and more effective pathway for the design of rare-earth oxide-based supercapacitor electrodes.

It should be noted that the proposed interaction between PAA functional groups and Nd^3+^ species is inferred from the observed concentration-dependent structural and electrochemical evolution rather than directly verified through surface-sensitive characterization techniques. Therefore, the polymer-assisted modulation mechanism discussed here should be regarded as a plausible interpretation based on indirect experimental evidence, including morphological variation, reduced charge-transfer resistance, and improved electrochemical kinetics.

## 4. Electrochemical Performance of Asymmetric Supercapacitor Device

To assess the practical viability of the optimized material beyond three-electrode measurements, an asymmetric supercapacitor device (ASD) was constructed by employing the Nd_2_O_3_-P2 electrode as the positive electrode and activated carbon (AC) as the negative electrode. The choice of AC is strategically justified by its well-known electric double-layer capacitive behavior, high surface area, and rapid charge–discharge capability, which effectively complement the Faradaic nature of Nd_2_O_3_ [46,47]. This complementary pairing enables a hybrid charge storage mechanism, integrating surface-driven capacitive effects with diffusion-mediated redox reactions to achieve enhanced overall device performance [48]. Both electrodes were fabricated on nickel foam substrates to ensure efficient current collection, low interfacial resistance, and mechanical robustness. The negative electrode was prepared via a conventional slurry-casting approach using activated carbon, conductive carbon, and a polymeric binder (80:10:10 ratio) dispersed in an appropriate solvent, followed by uniform coating and drying to achieve a stable electrode layer. Both electrodes were fabricated with identical geometric areas (1 × 1 cm^2^), and the device assembly was carried out using a practical balancing approach by controlling the mass loading of the AC electrode (~2 mg/cm^2^) and evaluating the electrochemical response to ensure stable and efficient operation. The assembled device utilized an aqueous alkaline electrolyte (2 M KOH) along with a porous separator to facilitate ionic conduction while preventing electrical contact between the electrodes. The asymmetric device assembly was guided according to the conventional charge balance principle (12) [46]:

(12) $$q^{+}=q^{-}$$

where the stored charge depends on capacitance, operating voltage window, and active material loading. In conventional slurry-based systems, this relationship is often used to determine the precise mass ratio between positive and negative electrodes. However, in the present work, the positive electrode was directly grown on nickel foam through an in situ hydrothermal route, resulting in a binder-free and substrate-integrated architecture. Under these conditions, accurate gravimetric determination of the deposited active material may involve uncertainty due to possible substrate variation during hydrothermal treatment and annealing. Therefore, the device assembly was practically optimized using identical geometric electrode areas together with electrochemical balancing considerations based on the operating potential range and charge-storage behavior of both electrodes.

**Figure 9:**
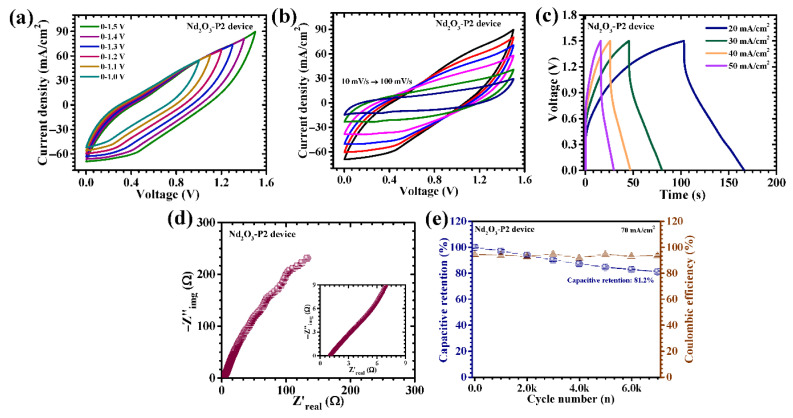
Performance of the assembled Nd_2_O_3_-P2//AC asymmetric supercapacitor: (**a**) CV curves at different voltage windows, (**b**) CV responses at varying scan rates (10–100 mV/s), (**c**) GCD profiles at multiple current densities (20–50 mA/cm^2^), (**d**) Nyquist plots, and (**e**) Cycling stability performance.

The electrochemical performance of the Nd_2_O_3_-P2//AC device was systematically evaluated using CV, GCD, and EIS. Initially, CV measurements were conducted over progressively widened voltage windows to establish the stable operating voltage range. The device maintains stable electrochemical behavior up to an extended voltage window of approximately 1.5 V without noticeable distortion or onset of parasitic reactions (Figure 9a), indicating good electrochemical stability. Further CV analysis performed at scan rates ranging from 10 to 100 mV/s (Figure 9b) reveals quasi-rectangular curves embedded with subtle redox features, confirming the coexistence of electric double-layer capacitance from the AC electrode and Faradaic redox activity from the Nd_2_O_3_ electrode. The preservation of the overall CV profile at higher scan rates indicates favorable reaction kinetics and efficient charge propagation within the device architecture. The GCD profiles recorded at varying current densities of 20 to 50 mA/cm^2^ (Figure 9c) exhibit non-linear charge–discharge characteristics with distinct voltage plateaus, further confirming the dominant contribution of Faradaic processes. The asymmetry observed in the charge–discharge curves reflect the hybrid nature of the device, combining capacitive and battery-type behavior. At a current density of 20 mA/cm^2^, the device delivers a notable areal capacitance of 0.3029 F/cm^2^ along with appreciable energy and power densities (Table 4), demonstrating a well-balanced energy–power output suitable for practical applications. Even at elevated current densities, the device retains a significant portion of its initial capacitance, highlighting its excellent rate capability. This behavior can be attributed to the optimized microstructure of the Nd_2_O_3_-P2 electrode, which facilitates rapid ion diffusion and efficient electron transport across the electrode–electrolyte interface.

Further insights into the internal resistance and charge transfer processes were obtained through EIS analysis. The Nyquist plot (Figure 9d) displays a small semicircle in the high-frequency region followed by a nearly linear response at low frequencies, indicating low charge transfer resistance and favorable ion diffusion characteristics. The extracted Rs is relatively low (1.04 Ω), reflecting efficient ionic and electronic conduction pathways within the device. The long-term cycling stability of the Nd_2_O_3_-P2//AC asymmetric device was evaluated through prolonged charge–discharge cycling up to 7000 cycles at a high current density of 70 mA/cm^2^ (Figure 9e). The device demonstrates excellent durability, retaining a high percentage of its initial capacitance of 81.2% even after thousands of cycles, accompanied by consistently high coulombic efficiency (93.6%), indicating highly reversible electrochemical behavior with minimal energy loss. The superior cycling stability can be attributed to the structurally robust and interconnected framework of the PAA-engineered Nd_2_O_3_ electrode, which effectively accommodates repeated volumetric changes associated with ion insertion and extraction without significant degradation. Meanwhile, the AC electrode maintains stable capacitive behavior, ensuring consistent performance throughout the cycling process. Overall, the Nd_2_O_3_-P2//AC asymmetric supercapacitor exhibits a compelling combination of an extended operating voltage window, high capacitance, favorable energy–power characteristics, and excellent long-term stability, demonstrating that PAA-assisted structural engineering of Nd_2_O_3_ effectively translates from material-level enhancement to superior device-level performance.

## 5. Conclusions

A systematic investigation was carried out to understand the influence of PAA on the structural and electrochemical properties of Nd_2_O_3_ electrodes. The results clearly show that PAA plays a critical role in controlling particle growth and surface morphology. The pristine Nd_2_O_3_ sample exhibits a highly agglomerated structure, which limits electrolyte access and reduces electrochemical activity. With the introduction of PAA, the morphology gradually evolves, and at an optimized concentration (Nd_2_O_3_-P2), a more uniform, porous, and interconnected structure is obtained. This optimized structure improves electrolyte penetration and facilitates ion transport, resulting in enhanced electrochemical performance. The Nd_2_O_3_-P2 electrode delivers the highest areal capacitance, along with better rate capability and lower internal resistance compared to other samples. It also shows good cycling stability, maintaining 87.08% of its capacitance after 12,000 cycles. The asymmetric device assembled using the optimized electrode operates stably up to 1.5 V and retains 81.2% capacitance after 7000 cycles, confirming its practical applicability. These results indicate that controlling the structure of Nd_2_O_3_ through PAA incorporation is an effective approach to improve its performance for supercapacitor applications.

## Figures and Tables

**Table 1:** Summary of electrochemical kinetic parameters including diffusion coefficients (D), b-values derived from CV analysis, and equivalent series resistance (Rs) for Nd_2_O_3_-based electrodes.

Sample Code	Diffusion Coefficient (cm^2^/s) × 10^−8^		b-Value	Rs (Ω)
	Oxidation	Reduction		
Nd_2_O_3_	0.7411	0.7835	0.56	1.05
Nd_2_O_3_-P1	10.08	7.535	0.52	0.72
Nd_2_O_3_-P2	24.047	14.184	0.51	0.67
Nd_2_O_3_-P3	6.507	4.717	0.47	0.82

**Table 2:** Calculated electrochemical performance metrics including areal capacitance (C_A_), areal capacity (C), energy density (ED), and power density (PD) at different applied current densities.

Sample Code	I (mA/cm^2^)	Areal Capacitance C_A_ (F/cm^2^)	Capacity (mAh/cm^2^)	Energy Density ED (mWh/cm^2^)	Power Density PD (mW/cm^2^)
Nd_2_O_3_	10	8.000	0.333	0.100	1.33
	20	7.333	0.306	0.092	2.83
	30	5.333	0.222	0.067	4.07
	40	4.444	0.185	0.056	5.33
	50	3.079	0.093	0.031	7.69
Nd_2_O_3_-P1	10	13.778	0.574	0.172	1.37
	20	12.000	0.500	0.150	2.69
	30	11.333	0.472	0.142	3.81
	40	8.889	0.370	0.111	4.85
	50	4.444	0.185	0.056	6.06
Nd_2_O_3_-P2	10	26.889	1.120	0.336	1.25
	20	22.667	0.944	0.283	2.39
	30	20.000	0.833	0.250	3.50
	40	12.444	0.519	0.156	4.38
	50	6.667	0.278	0.083	5.00
Nd_2_O_3_-P3	10	12.222	0.509	0.153	1.38
	20	12.000	0.500	0.150	2.71
	30	10.667	0.444	0.133	3.98
	40	7.111	0.296	0.089	4.72
	50	3.415	0.105	0.046	5.63

**Table 3:** Comparative analysis of electrochemical performance parameters of previously reported Nd_2_O_3_-based oxide electrode systems.

Sr. No.	Material	Synthesis Method	Current	Electrolyte	Areal Capacitance	Cycle Stability	Ref.
1.	Nd_2_O_3_/Co_3_O_4_/rGO/NF	Hydrothermal/ Annealing process	1 A/g	6 M KOH	3359.6 F/g	30,000 cycles (95.7% stability)	[28]
2.	CuCo(5-NIPA)–Nd_2_O_3_	Sonication	2.1 A/g	-	5.06 F/cm^2^	3000 cycles (93.4% stability)	[33]
3.	Nd_2_O_3_/Sm_2_O_3_	Sol–gel	2 A/g	2 M KOH	2059 F/g	5000 cycles	[44]
4.	Nd-doped Mn_2_O_3_	Hydrothermal	0.5 A/g	1 M KOH	4.01 F/cm^2^	2000 cycles (97.3% stability)	[45]
5.	Nd_2_O_3_-V_2_O_5_	solution combustion method	1 A/g	-	813 F/g	5000 cycles (95.3% stability)	[40]
6.	PPy/Nd_2_O_3_	Electrodeposition	1 A/g	0.1 M H_2_SO_4_	268 F/g	3000 cycles (88% stability)	[13]
7.	PAA-Nd_2_O_3_	Hydrothermal	10 mA/cm^2^	2 M KOH	26.889 F/cm^2^	12,000 cycles (87.08% stability)	This work

**Table 4:** Device-level energy storage performance parameters of the Nd2O3-P2//AC asymmetric supercapacitor.

Sample Code	I (mA)	Areal Capacitance C_A_ (F/cm^2^)	Capacity (mAh/cm^2^)	Energy Density ED (mWh/cm^2^)	Power Density PD (mW/cm^2^)
Nd_2_O_3_-P2 device	20	0.302	0.063	0.094	2.70
	30	0.240	0.050	0.075	3.86
	40	0.178	0.037	0.056	4.76
	50	0.133	0.028	0.042	5.36

## Data Availability

The original contributions presented in this study are included in the article. Further inquiries can be directed to the corresponding author.
